# Broken Engines of Immunity: Mitochondrial Dysfunction in T Cells Across Cancer and Complex Pathologies

**DOI:** 10.1002/eji.70300

**Published:** 2026-10-28

**Authors:** Carolina Brescia, Giuseppe Viglietto, Rosario Amato

**Affiliations:** ^1^ Medical Genetics Unit Department of Health Science Medical School University Hospital “Mater Domini” University “Magna Graecia” Catanzaro Italy; ^2^ Immunogenetics Laboratory Department of Health Science Medical School University “Magna Graecia” Catanzaro Catanzaro Italy; ^3^ Department of Experimental and Clinical Medicine “G. Salvatore” Medical School University “Magna Graecia” Catanzaro Catanzaro Italy

**Keywords:** cancer, metabolism, mitochondria, mitochondrial dysfunction, T cell, T‐cell exhaustion

## Abstract

Mitochondria have traditionally been viewed as the cellular ‘powerhouses’ responsible for energy production; however, they also play essential roles in a wide range of cellular processes, including macromolecule biosynthesis and the regulation of apoptosis. Accumulating evidence highlights their importance in immune regulation, particularly through their influence on T cell activation, proliferation, and effector function. In addition to ATP generation, mitochondria regulate redox and calcium homeostasis and produce metabolites that modulate the transcriptional and epigenetic programs of immune cells. Upon antigen recognition, T lymphocytes undergo marked metabolic reprogramming, characterised by increased glycolysis while preserving mitochondrial metabolism as a critical pathway. Mitochondrial dysfunction disrupts intracellular signalling and contributes to T cell exhaustion in diverse pathological contexts, including cancer and chronic viral infections. Consequently, strategies aimed at restoring mitochondrial function are emerging as promising therapeutic approaches to correct immune dysfunction driven by impaired T cell responses. Importantly, these alterations should not be interpreted solely as downstream consequences of chronic immune stimulation; rather, mitochondrial dysfunction can actively determine T‐cell fate, stabilise exhaustion‐associated programs, and define the extent to which dysfunctional T cells remain therapeutically reversible. This review first outlines mitochondrial structure, bioenergetics, dynamics, and quality‐control pathways and then examines how these processes are remodelled across naïve, effector, circulating‐memory, tissue‐resident‐memory, and exhausted T‐cell states. It subsequently integrates evidence from cancer, chronic viral infection, and ageing and evaluates mitochondria‐directed strategies for restoring T‐cell function.

## Graphical abstract

Mitochondria not only play a fundamental role in energy production but also regulate physiological processes, including metabolism, homeostasis, and immune function. In T cells, they control activation, differentiation, and function. Their dysfunction contributes to immune exhaustion in cancer, chronic infections, and ageing. This review examines these mechanisms and mitochondrial‐targeted therapies.

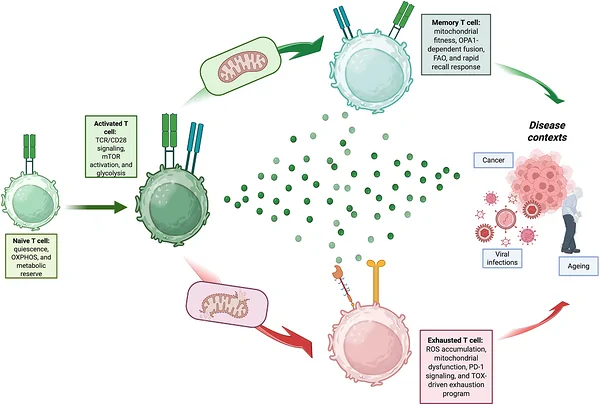

## 1 Introduction

At the interface between cellular metabolism and immune signalling, mitochondria occupy a central position in T‐cell biology, not simply as sources of ATP but as organelles that connect nutrient availability with biosynthesis and gene regulation [1]. Their contribution to immune function extends beyond oxidative phosphorylation: mitochondrial redox activity, calcium handling and the production of metabolic intermediates influence the transcriptional and epigenetic programmes that govern T‐cell activation, survival and differentiation (Table 1) [2, 3]. These functions depend on close coordination between nuclear‐ and mitochondrial‐encoded components and on an organellar architecture that is continuously remodelled to match cellular demand [2, 3, 4]. This plasticity becomes particularly important after antigen recognition. T‐cell receptor and co‐stimulatory signals trigger a rapid increase in nutrient uptake and a broad metabolic reprogramming controlled in large part by mTOR‐ and AMPK‐centred pathways (Figure 1). Activated effector T cells increase aerobic glycolysis to support rapid proliferation and cytokine production; however, mitochondrial metabolism remains essential for signalling competence, biosynthetic reactions and the establishment of long‐lived or regulatory states [5]. Consequently, glycolysis and mitochondrial respiration should not be regarded as mutually exclusive programmes. Instead, the relative contribution of these cells varies across naïve, effector, regulatory, and memory T‐cell populations (Table 1). Quiescent naïve cells exhibit comparatively modest energetic demands. Rapidly expanding effectors prioritise pathways that provide ATP and biosynthetic precursors. Long‐lived memory cells retain substantial mitochondrial reserve and metabolic flexibility. In addition, tissue‐resident populations must adapt to the oxygen tension and nutrient composition of their local niche. Mitochondrial morphology and quality control represent an additional regulatory layer: The preservation of respiratory capacity and metabolic flexibility is achieved by DRP1‐dependent fission, MFN1/MFN2‐ and OPA1‐dependent fusion, PGC‐1α‐driven biogenesis and selective removal of damaged organelles [6, 7, 8]. In instances where these adaptive mechanisms are compromised, mitochondria can become a significant contributor to the onset of T‐cell dysfunction. Impaired biogenesis, altered fission–fusion balance, defective quality control and excessive reactive oxygen species (ROS) accumulation have all been linked to progressive loss of T‐cell function during chronic antigen exposure. In cancer and persistent viral infection, these abnormalities reinforce exhaustion‐associated transcriptional and epigenetic programmes and may limit the capacity of dysfunctional cells to respond to therapeutic reinvigoration [9, 10, 11, 12, 13]. Mitochondrial fitness is therefore not merely a readout of activation history; it can influence T‐cell fate, persistence and functional reversibility. Against this background, the following section provides a focused overview of the mitochondrial features required to understand T‐cell biology, before examining their role in activation, differentiation and disease and discussing mitochondria‐directed therapeutic strategies.

**TABLE 1:** Mitochondrial regulation of T‐cell function and dysfunction.

Mitochondrial axis	Key molecular components	Physiological role in T cells	Consequence of dysfunction	Potential therapeutic approaches
Energy metabolism	OXPHOS, ETC complexes, TCA cycle, ATP synthase	Supports ATP production, survival, activation, proliferation, and memory T‐cell maintenance.	Reduced ATP availability, impaired proliferation, defective effector function, and loss of metabolic fitness.	Enhancement of mitochondrial respiration and biogenesis; metabolic support of memory and effector T‐cell subsets.
Redox homeostasis	Mitochondrial ROS, SOD2, GPX, catalase, NAD^+^/NADH balance	Provides controlled redox signalling required for TCR activation and downstream NFAT, NF‐kB, and AP‐1 signalling.	Excess ROS, oxidative damage, impaired TCR signalling, apoptosis, and stabilisation of exhaustion‐associated programs.	Antioxidant strategies, ROS scavengers, mitochondria‐targeted antioxidants, and NAD^+^ restoration.
Calcium handling	MCU complex, MICU1/2, NCLX, mitochondrial membrane potential	Couples mitochondrial activity to Ca^2+^‐dependent signalling, activation, and survival.	Defective Ca^2+^ flux, altered activation thresholds, impaired survival, and reduced signalling competence.	Modulation of mitochondrial Ca^2+^ uptake and release; restoration of mitochondrial membrane potential.
Mitochondrial dynamics and quality control	DRP1, MFN1, MFN2, OPA1, PINK1, Parkin, mitophagy pathways	Maintains mitochondrial morphology, content exchange, organelle quality, and adaptation to activation or stress.	Fragmentation or pathological hyperfusion, accumulation of damaged mitochondria, reduced respiratory efficiency, and T‐cell exhaustion.	Promotion of balanced fission/fusion, enhancement of mitophagy, and support of mitochondrial quality control.
Mitochondrial biogenesis	PGC‐1alpha, AMPK, mTOR, IL‐15‐associated signalling	Expands mitochondrial mass and respiratory capacity; supports memory formation and long‐term T‐cell persistence.	Reduced mitochondrial mass, impaired metabolic plasticity, defective memory formation, and reduced reversibility of exhaustion.	Activation of PGC‐1alpha‐dependent biogenesis; AMPK modulation; IL‐15, metformin, bezafibrate, or combination with checkpoint blockade.
Metabolite‐dependent gene regulation	Acetyl‐CoA, alpha‐ketoglutarate, succinate, NAD^+^, SAM, lactate	Links mitochondrial metabolism to chromatin accessibility, histone modifications, and T‐cell differentiation.	Epigenetic reprogramming, altered Th17/Treg balance, and stabilisation of dysfunctional or exhausted phenotypes.	Targeting metabolic checkpoints; restoration of NAD^+^ availability; modulation of metabolite‐dependent epigenetic pathways.
Disease‐associated mitochondrial dysfunction	Cancer, chronic viral infection, ageing, chronic inflammation	Preserves immune competence, effector function, and memory responses under stress.	T‐cell exhaustion, senescence, impaired antiviral or antitumour immunity, and reduced response to immunotherapy.	Mitochondria‐targeted therapies, checkpoint blockade combinations, mitochondrial transfer, and metabolic reprogramming strategies.

*Note*: Major mitochondrial pathways and their contribution to T‐cell activation, differentiation, exhaustion, and therapeutic reversibility are summarised.

Abbreviations: AMPK, AMP‐activated protein kinase; AP‐1, activator protein 1; DRP1, dynamin‐related protein 1; ETC, electron transport chain; GPX, glutathione peroxidase; MCU, mitochondrial calcium uniporter; MFN, mitofusin; MICU, mitochondrial calcium uptake protein; mTOR, mechanistic target of rapamycin; NAD^+^, nicotinamide adenine dinucleotide; NCLX, mitochondrial Na^+^/Ca^2+^ exchanger; NFAT, nuclear factor of activated T cells; NF‐kB, nuclear factor kB; OPA1, optic atrophy protein 1; OXPHOS, oxidative phosphorylation; PGC‐1alpha, peroxisome proliferator‐activated receptor gamma coactivator 1‐alpha; ROS, reactive oxygen species; SAM, S‐adenosylmethionine; SOD2, superoxide dismutase 2; TCA, tricarboxylic acid; TCR, T‐cell receptor.

**FIGURE 1:**
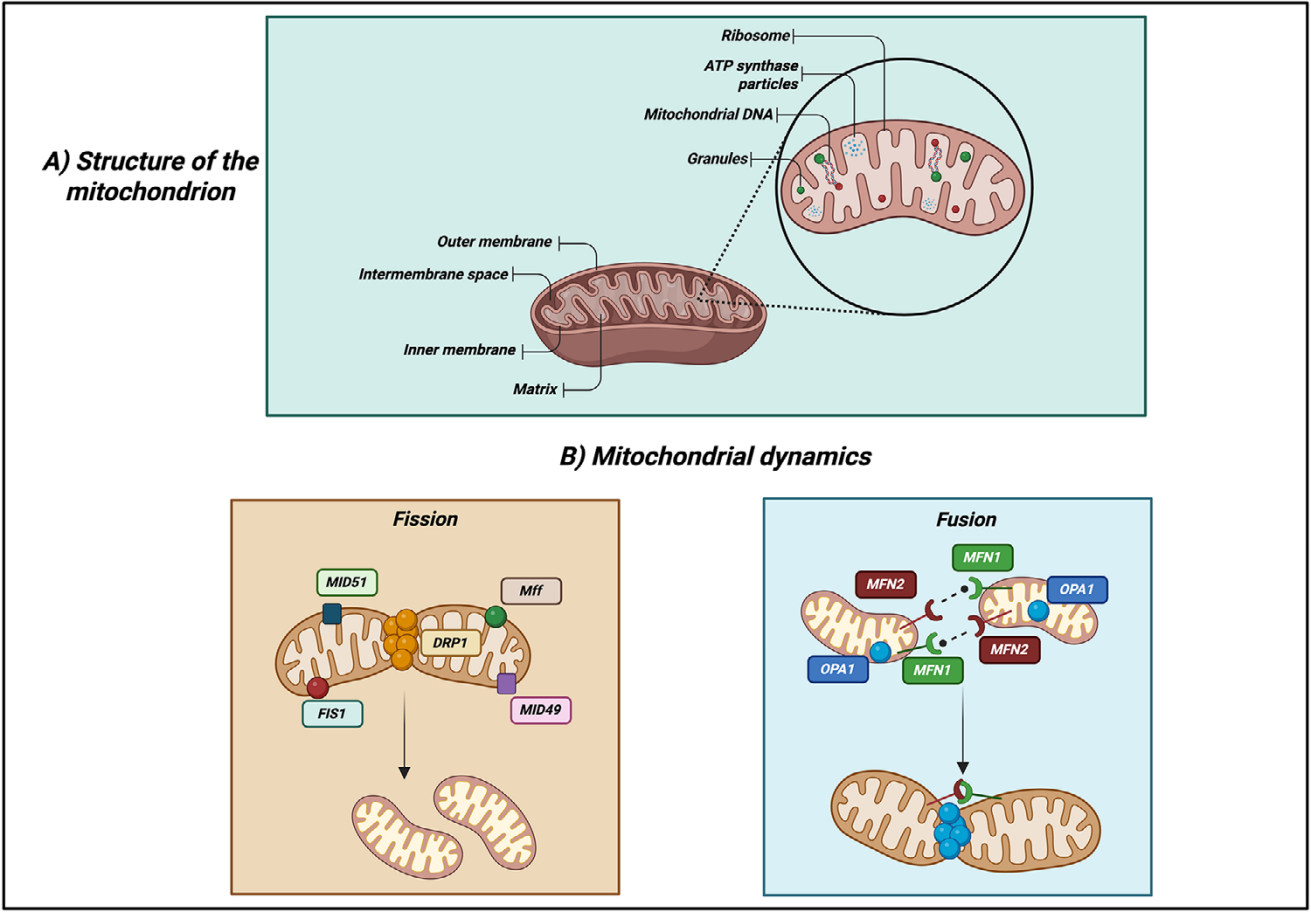
Mitochondrial structure and dynamics. (A) Mitochondrial structure: illustration of the main mitochondrial components, including mitochondrial DNA, ATP synthase particles, ribosomes and matrix granules, as well as the outer mitochondrial membrane, the inner membrane and the mitochondrial matrix, which are essential for the energy and metabolic function of the mitochondrion. (B) Mitochondrial fusion and fission: the image illustrates the dynamic processes that regulate the shape, distribution and function of mitochondria. Created in BioRender. *Source*: Brescia, C. (2026) https://BioRender.com/gi0tun6.

## 2 Mitochondria: Structure and Function

Within T cells, the mitochondrial compartment coordinates energy production with biosynthesis, redox control, calcium signalling and cell‐death pathways [4]. Oxidation of pyruvate, fatty acids and amino acids generates acetyl‐CoA, which enters the tricarboxylic acid (TCA) cycle in the mitochondrial matrix. The resulting NADH and FADH2 supply electrons to the electron transport chain (ETC) in the inner mitochondrial membrane, establishing the proton gradient that drives ATP synthesis through oxidative phosphorylation (OXPHOS) [1]. ATP is subsequently exchanged across the inner membrane through the adenine nucleotide translocator and reaches the cytosol through voltage‐dependent anion channels (VDACs), linking mitochondrial output to cellular energy demand [14].

The TCA cycle also provides intermediates for lipid, amino‐acid and nucleotide synthesis, allowing mitochondria to support biomass accumulation as well as ATP production [2]. At the same time, mitochondrial metabolism helps maintain the NAD^+^/NADH and NADP^+^/NADPH balance and generates controlled amounts of ROS through electron transfer in the ETC. At physiological levels, ROS participate in signalling; when production exceeds antioxidant capacity, however, they damage proteins, lipids and nucleic acids and compromise organelle function [15]. Mitochondria also shape intracellular Ca^2+^ signals by taking up and releasing calcium, thereby coupling receptor signalling to metabolic activity [16]. Under severe stress, mitochondrial outer‐membrane permeabilisation, mediated principally by the pro‐apoptotic BCL‐2‐family proteins BAX and BAK, releases cytochrome c and other apoptogenic factors, including SMAC/DIABLO, AIF and EndoG, to initiate intrinsic apoptosis [17, 18, 19]. These interconnected functions are directly relevant to T cells, in which activation, proliferation, survival and contraction all require tight coordination between metabolic supply and signalling demand. Within the context of T‐lymphocytes, the regulatory mechanisms governing mitochondrial apoptosis entail a delicate balance between pro‐survival BCL‐2‐family proteins and pro‐apoptotic BH3‐only proteins. These proteins act as stress sensors, modulating the activation of BAX/BAK‐mediated mitochondrial outer‐membrane permeabilisation in response to declining cytokine‐ or antigen‐derived survival signals [20, 21]. The BH3‐only protein BIM plays a pivotal role during immune‐response contraction. Bim deficiency has been demonstrated to hinder the physiological deletion of antigen‐specific CD8^+^ T cells following clearance of a viral infection. In doing so, this deficiency establishes a direct link between mitochondrial apoptosis and T‐cell homeostasis [22].

### 2.1 Genome and Mitochondrial Structure

Although human mitochondria retain a small circular genome of bacterial origin, their function depends predominantly on the nuclear genome. Mitochondrial DNA (mtDNA) encodes 13 OXPHOS subunits, 22 transfer RNAs and two ribosomal RNAs, whereas more than 99% of mitochondrial proteins are nuclear‐encoded and imported through the translocase of the outer and inner membrane complexes (TOM and TIM) [23, 24]. This division of genetic control requires continuous communication between mitochondria, the nucleus and the cytoplasm. mtDNA is organised into nucleoids associated with the inner membrane rather than with histones, and its maintenance is influenced by replication, repair, turnover and exposure to mitochondria‐derived ROS [25, 26]. Unlike the diploid nuclear genome, mtDNA is present in tens to thousands of copies per cell. This high copy number makes heteroplasmy, the coexistence of distinct mitochondrial genomes within the same cell, a common feature of mitochondrial genetics. The biological consequences of a variant depend on its abundance, tissue distribution and functional impact, rather than on the presence of the mutation alone. When the proportion of functionally impaired genomes rises beyond a tissue‐specific threshold, respiratory capacity can decline. Accordingly, accumulation of mtDNA damage or pathogenic variants has been implicated in inherited mitochondrial disorders as well as in cancer, ageing‐related conditions and neurodegenerative disease [18, 19, 24, 26, 27]. In immune cells, changes in mtDNA integrity or copy number are also relevant because they can alter respiratory fitness and promote the release of mitochondrial nucleic acids that act as inflammatory signals, concepts discussed in the pathological sections of this review. Mitochondrial architecture is equally important for function (Figure 1). The outer mitochondrial membrane separates the organelle from the cytosol and contains channels and protein‐import machinery that regulate metabolite exchange, organelle communication and apoptotic signalling [28, 29, 30]. The inner membrane forms cristae, which expand the surface available for ETC complexes and ATP synthase and help organise respiratory supercomplexes. The electrochemical gradient across this membrane is sustained by the relatively alkaline matrix and is central to ATP generation. The intermembrane space contains factors involved in protein import and apoptosis, whereas the matrix houses mtDNA, mitochondrial ribosomes and the enzymes responsible for the TCA cycle and other metabolic pathways [28, 29]. Cristae organisation and membrane integrity are therefore not merely structural properties: they determine respiratory efficiency, metabolite exchange and the ability of mitochondria to support rapidly changing cellular programmes. This is particularly relevant in T cells, where activation and differentiation are accompanied by marked changes in mitochondrial mass, ultrastructure and respiratory capacity.

### 2.2 Mitochondrial Dynamics

Continuous remodelling of shape, abundance and intracellular distribution allows the mitochondrial network to adjust to changing cellular demands through coordinated cycles of fission and fusion (Figure 1) [6, 28]. Fission divides a mitochondrion into daughter organelles and facilitates redistribution, segregation of damaged regions and organelle turnover. It commonly occurs at endoplasmic‐reticulum contact sites, where adaptor proteins including Fission protein 1 (Fis1), mitochondrial fission factor (Mff), and mitochondrial dynamics proteins of 49 and 51 kDa (MiD49 and MiD51) recruit the cytosolic GTPase dynamin‐related protein 1 (DNM1L/DRP1). DRP1 oligomerisation constricts the mitochondrial membranes, and its activity is tuned by post‐translational modifications such as phosphorylation, SUMOylation and ubiquitination [28, 31, 32]. Beyond mitochondrial distribution, fission contributes to cell‐cycle progression, stress responses and apoptosis [7, 33]. Fusion proceeds through coordinated merging of the outer and inner mitochondrial membranes and permits mixing of membrane components, metabolites and mitochondrial genomes [6, 34]. Mitofusin 1 (MFN1) and mitofusin 2 (MFN2) mediate outer‐membrane fusion, whereas OPA1 controls inner‐membrane fusion and supports cristae organisation [28, 35, 36, 37]. The interconnected networks generated by fusion can improve respiratory efficiency and enable functional complementation between mitochondrial compartments, thereby increasing tolerance to local damage or mtDNA defects [34, 38]. The balance between fission and fusion is therefore functional rather than purely morphological: fragmented and fused networks differ in substrate use, respiratory organisation and susceptibility to stress. These processes are integrated with mitochondrial quality control. In the event of damaged or depolarised mitochondria, a process referred to as mitophagy can be initiated, whereby the affected mitochondria are selectively removed. This process is most notably facilitated by the PINK1–Parkin pathway. Concurrently, PGC‐1α‐dependent mitochondrial biogenesis is initiated, with the objective of replenishing the network and restoring the capacity of the organelles [7, 28, 39]. These pathways function in a collaborative manner: the process of fission has been shown to separate regions of the mitochondrion that are not functioning optimally. In contrast, fusion has been observed to act as a form of temporary buffering against defects in specific regions through the mixing of constituent components. Mitochondrial transportation further positions the organelles at locations where there is local energetic and signalling demand, a requirement that is especially evident in highly polarised cells [40]. Failure of mitophagy, transport or fission–fusion control leads to the accumulation of dysfunctional mitochondria and is associated with diverse pathological states [40, 41]. In T cells, the same quality‐control machinery supports the rapid transition from quiescence to proliferation and later contributes to memory formation and persistence. Conversely, chronic disruption of mitochondrial dynamics and turnover can amplify oxidative stress and metabolic insufficiency, providing a mechanistic bridge between organelle damage and the exhaustion programmes discussed below.

## 3 Mitochondria as Key Regulators in T cell Activation and Function

In recent years, numerous studies have demonstrated that mitochondria play an essential role in regulating inflammatory processes and shaping subsequent immune responses [42]. Through the release of pro‐inflammatory signals, including reactive oxygen species (ROS) and mitochondrial‐derived components that function as damage‐associated molecular patterns (DAMPs), mitochondria act as key modulators of inflammation, thereby influencing immune activation and, in particular, T lymphocyte responses [9, 42]. Consistent with this, accumulating evidence indicates that mitochondria are crucial regulators of T lymphocyte activation and metabolic reprogramming processes that are fundamental for effective immune function [9] (Figure 2). From this perspective, mitochondria should be considered active signalling platforms capable of directing immune trajectories rather than passive organelles that simply adapt to pre‐existing activation states.

**FIGURE 2:**
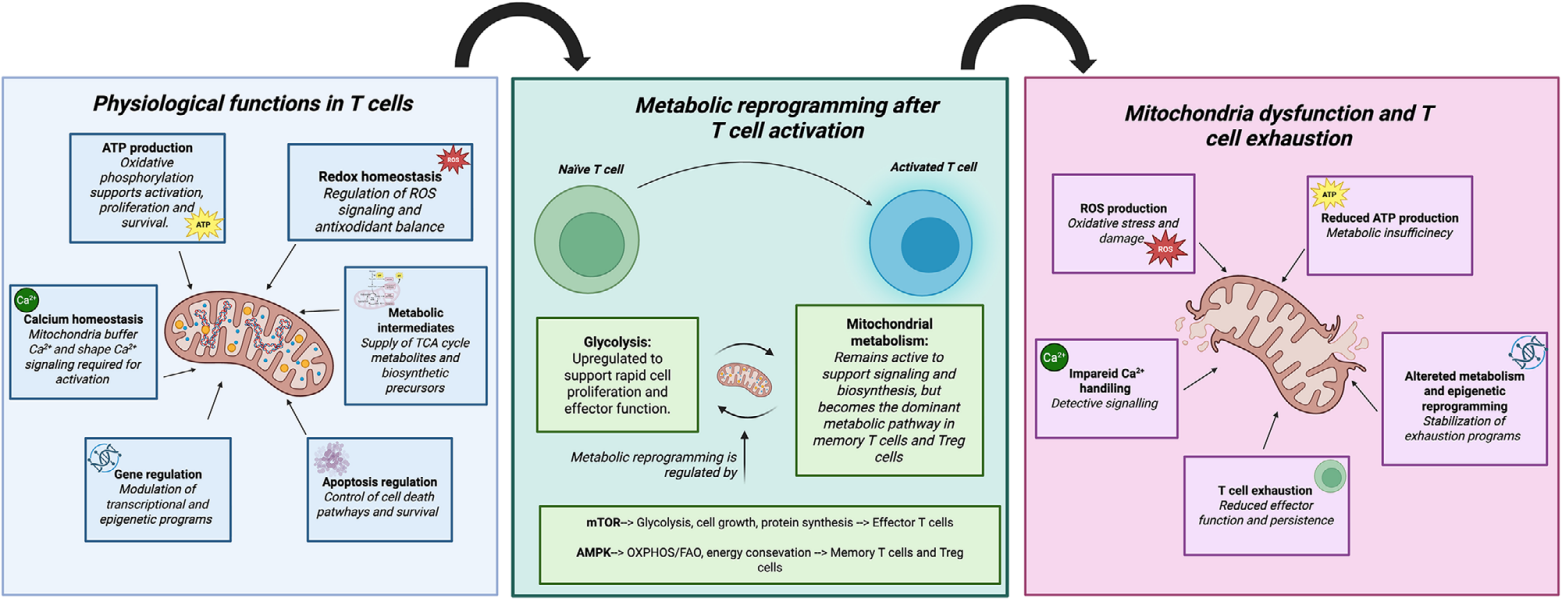
Mitochondrial function in T cells. The figure illustrates how mitochondrial function varies across the different stages of T‐cell development, from naïve T cells to activated and exhausted T cells. In addition, it highlights the various cellular processes that undergo upregulation or downregulation. Created in BioRender. *Source*: Brescia, C. (2026) https://BioRender.com/o1mcrvl.

### 3.1 T‐Cell Differentiation States and Functional Subsets

T cells comprise phenotypically and functionally distinct populations that arise through antigen‐ and context‐dependent differentiation. Naïve CD4^+^ and CD8^+^ T cells recirculate through secondary lymphoid organs and, after T‐cell receptor engagement, co‐stimulation, and cytokine exposure, undergo clonal expansion and acquire effector functions. CD4^+^ cells differentiate into helper and regulatory lineages, including Th1, Th2, Th17, T follicular helper, and Treg cells [5, 43, 44], whereas activated CD8^+^ T cells become cytotoxic effectors [44]. After antigen clearance, most effector cells die during the contraction phase, while a fraction persists as memory cells with enhanced recall capacity [8, 45, 46]. Memory is itself heterogeneous. Central memory T cells (TCM) express lymphoid‐homing receptors such as CCR7 and CD62L and retain high proliferative potential; effector memory T cells (TEM) circulate through blood and peripheral tissues and provide rapid effector activity; and tissue‐resident memory T cells (TRM), often identified by CD69 and, in epithelial sites, CD103, remain lodged within tissues and provide local immune surveillance. In contrast, persistent antigen exposure in cancer and chronic infection diverts differentiation toward a progenitor‐exhausted compartment (Tpex) that sustains the response and a terminally exhausted compartment with restricted proliferative and effector capacity [47, 48, 49, 50, 51]. The characterisation of these states does not depend solely on the phenotype; rather, each state is supported by a distinct balance of nutrient use, mitochondrial architecture, respiratory capacity, and redox signalling. This suggests that metabolism plays an integral role in T‐cell differentiation.

### 3.2 Energy Metabolism of T Cells

Naïve T lymphocytes are characterised by low energy requirements. They are dependent primarily on oxidative phosphorylation (OXPHOS) for the production of energy, a reflection of their quiescent state (Table 1). Upon antigen recognition through the T‐cell receptor (TCR) and engagement of co‐stimulatory receptors such as CD28, T cells become activated and differentiate into effector subsets, including Th1, Th2, Th17, and CD8^+^ effector T cells [45, 46, 52]. Seminal studies established that CD28 co‐stimulation is metabolically instructive rather than merely permissive: CD28 signalling increases GLUT1 expression and cell‐surface trafficking, enhances glucose uptake and sustains glycolytic flux through both Akt‐dependent and Akt‐independent pathways [53, 54]. These findings provided early evidence that co‐stimulatory signals directly couple antigen recognition to the nutrient acquisition required for T‐cell growth, IL‐2 production and clonal expansion. Activated T cells consequently face a rapid increase in bioenergetic and biosynthetic demands that support proliferation, cytokine production and effector function. To meet these demands, they shift towards aerobic glycolysis even in the presence of oxygen (Table 1). This phenomenon, termed the Warburg effect, has garnered significant scientific interest [45, 46, 47]. During this metabolic reprogramming, activated effector T cells have been observed to increase glucose uptake and catabolism, while also utilizing amino acids such as glutamine to generate pyruvate and lactate, thereby reducing their reliance on mitochondrial respiration (43). The transcription factors c‐MYC and hypoxia‐inducible factor‐1α (HIF‐1α) play central roles in this process by regulating the expression of glycolytic genes, including lactate dehydrogenase A (LDHA) and glucose transporters GLUT1–4 [8]. A key upstream regulator of this transcriptional program is mechanistic target of rapamycin complex 1 (mTORC1), which is essential for the activation of both HIF‐1α and c‐MYC and for the broader control of T‐cell metabolism and function [55]. In the context of CD4^+^ T‐cell differentiation, HIF‐1α plays a pivotal role by promoting Th17 lineage commitment through the induction of IL‐17A expression and by suppressing FOXP3 and regulatory T (Treg) cell differentiation [50]. Conversely, c‐MYC has been identified as being imperative for the proliferation and expansion of activated T cells, coordinating glycolysis, glutaminolysis, and the biosynthesis of nucleotides, lipids, and polyamines [51]. Amino acid metabolism has also been demonstrated to play a pivotal role in these metabolic adaptations [52]. Glutamine is a pivotal nutrient for activated T cells, as it is transformed by glutaminase into glutamate and subsequently into α‐ketoglutarate, an intermediate in the tricarboxylic acid (TCA) cycle that facilitates ATP synthesis [8]. This metabolic pathway is characteristic of rapidly proliferating cells, including activated immune cells. In addition, glutamine facilitates leucine uptake via the SLC7A5 transporter, thereby promoting mTORC1 activation [56], and favours differentiation toward Th1, Th17, and CD8^+^ effector phenotypes [8]. Asparagine also plays a significant role in T‐cell metabolism and function. In CD8^+^ T cells, asparagine binds the tyrosine kinase LCK, enhancing T‐cell activation and effector function. Increased extracellular asparagine levels improve CD8^+^ T‐cell responses both in vitro and in vivo, whereas dietary restriction or inhibition of the SLC1A5 transporter, which mediates asparagine uptake, significantly impairs CD8^+^ T‐cell activity [57]. The enhanced uptake of glucose and amino acids following T‐cell activation is enabled by the increased surface expression of specific transporters, including GLUT1, SLC7A5, and SLC1A5 [8, 56]. GLUT1 is particularly critical for effector T‐cell expansion, especially in CD4^+^ T cells, and for cytokine production. Elevated GLUT1 expression promotes Th17 differentiation at the expense of Treg development [5, 8]. Enhanced glycolytic flux in effector T cells also induces the expression of enzymes that exert both metabolic and epigenetic functions, thereby influencing gene expression [58]. LDHA, a key enzyme that catalyses the conversion of pyruvate to lactate while regenerating NAD^+^ to sustain glycolysis, is highly expressed in activated CD8^+^ T cells and Th17 cells and is essential for their effector functions. LDHA expression is regulated by mTOR, c‐MYC, and HIF‐1α during T‐cell activation [59]. The lactate produced can further modulate gene expression through histone lactylation, an epigenetic modification that regulates effector genes such as IFNG [60, 61]. In pathological settings, such as the tumour microenvironment, competition for metabolic substrates between tumour cells and effector T cells can impair T‐cell metabolism and compromise immune function [62]. Nucleotide metabolism is also indispensable for T‐cell activation [63]. Activated T cells require increased synthesis of purines and pyrimidines to support DNA replication during proliferation and RNA production for cytokine and receptor expression. Accordingly, the pentose phosphate pathway (PPP) is as critical as glycolysis in activated T cells [64]. Nucleotides can be generated through de novo synthesis or salvage pathways, with T lymphocytes relying predominantly on the de novo pathway [63]. Following antigen clearance, effector T cells either undergo apoptosis or differentiate into long‐lived memory T cells [8]. Memory T cells depend primarily on mitochondrial metabolism and fatty acid oxidation to meet their energetic demands, a process regulated by AMP‐activated protein kinase (AMPK). AMPK activation suppresses energy‐consuming pathways, inhibits mTOR signalling, and reduces the production of cytokines such as interferon‐γ (IFN‐γ) (Figure 2) [65, 66]. AMPK also promotes Treg differentiation by inhibiting GLUT1 expression and enhancing FOXP3 induction [66]. Collectively, these findings highlight how amino acids, metabolites such as lactate, and metabolic signalling pathways exert multifaceted control over T‐cell function by influencing epigenetic regulation, post‐translational modifications, nucleotide biosynthesis, and redox homeostasis.

### 3.3 Mitochondrial Specialisation of Circulating and Tissue‐Resident Memory T Cells

The differentiation between circulating memory T cells and those that are resident within the tissues is accompanied by divergent mitochondrial prerequisites. The circulation of memory populations, notably TCM cells, is a recurrent phenomenon that involves the continuous transit between lymphoid organs, blood, and peripheral tissues. This dynamic process necessitates the maintenance of metabolic flexibility, substantial spare respiratory capacity, and the capacity to expeditiously augment ATP production following restimulation. In CD8^+^ memory cells, mitochondrial fusion and OPA1‐dependent cristae organisation promote efficient electron‐transport‐chain assembly, oxidative phosphorylation, and fatty‐acid oxidation. In contrast, effector cells exhibit more fragmented mitochondria, which support glycolytic metabolism. The experimental enforcement of fusion in effector T cells has been demonstrated to impose memory‐like metabolic and functional features, thereby demonstrating that fission–fusion dynamics participate directly in fate determination, rather than merely reflecting activation status [67]. Furthermore, the presence of circulating memory CD8^+^ T cells has been demonstrated to facilitate fatty‐acid oxidation through a cell‐intrinsic lipid cycle. Rather than depending primarily on the direct uptake of long‐chain fatty acids, the synthesis of lipids is achieved via the use of extracellular glucose. The mobilisation of stored fatty acids is then initiated through lysosomal acid lipase in order to fuel mitochondrial oxidation. This cycle of lipid synthesis and lipolysis appears to be futile, yet it is energetically costly. However, it provides a rapidly accessible and tightly controlled substrate reservoir that supports memory formation and long‐term fitness [68]. The issue of permanent tissue residence presents a distinct set of constraints. TRMs do not have the capacity to readily migrate to nutrient‐rich compartments; instead, they are compelled to acclimatise to the oxygen tension and substrate composition of their immediate environment. In the skin, the expression levels of FABP4 and FABP5 are increased by CD8^+^ TRM cells, which then acquire exogenous free fatty acids and use mitochondrial β‐oxidation to support long‐term survival and protective function. The loss of this pathway compromises persistence [69]. Consequently, migratory memory cells emphasise systemic metabolic flexibility and cell‐intrinsic fuel management, while resident memory cells couple mitochondrial fitness to tissue‐specific nutrient acquisition. It is important to note, however, that this distinction is likely to vary among organs. Therefore, when designing metabolic interventions, it would be advisable to take this into consideration. This is because a strategy that benefits circulating memory cells may not reproduce the substrate requirements of a resident population.

### 3.4 Mitochondrial Function in T Cells: From Metabolic Support to Mitochondrial Dysfunction

As discussed above, naïve T lymphocytes are maintained in a quiescent state and rely predominantly on oxidative phosphorylation (OXPHOS) for energy production (Table 1). Upon activation, T cells undergo metabolic reprogramming, shifting toward glycolysis to rapidly generate ATP and support proliferation and effector functions (Table 1) [8]. Nevertheless, OXPHOS remains critically important during T‐cell activation, particularly for specific subsets such as regulatory T (Treg) cells and memory T cells, which depend on mitochondrial respiration to sustain long‐term energy requirements [8]. Oxidative phosphorylation is also essential for Th17 lymphocytes; although these cells preferentially utilise aerobic glycolysis, mitochondrial metabolism remains necessary as it regulates the activation of transcription factors involved in Th17 differentiation, including basic leucine zipper ATF‐like transcription factor (BATF) [70]. The tricarboxylic acid (TCA) cycle, which takes place within mitochondria, is another key metabolic pathway not only for generating reducing equivalents required for ATP production but also for producing metabolic intermediates essential for protein and lipid biosynthesis and T ‐cell function, such as α‐ketoglutarate and acetyl‐CoA [71, 72]. These metabolites also play central roles in epigenetic regulation, as they directly or indirectly influence histone methylation and acetylation, thereby shaping T‐cell differentiation and function (Table 1) [71, 73]. Dysregulation of mitochondrial metabolism and excessive accumulation of these intermediates can lead to phenotypic alterations; for example, increased α‐ketoglutarate levels have been shown to reduce FOXP3 expression, thereby limiting Treg polarisation [72]. In the context of Th17 cells, mitochondrial dynamics are equally crucial. A loss of OPA1 impedes the tricarboxylic acid (TCA) cycle and the Th17 differentiation process, leading to defective effector function. In this particular setting, liver kinase B1 (LKB1) functions as an energy stress sensor, thereby restraining Th17 effector activity in response to mitochondrial dysfunction, as exemplified by OPA1 loss [67]. During the activation process, T lymphocytes have been observed to increase flux through the pentose phosphate pathway (PPP), which is essential for the synthesis of nucleotides necessary for DNA replication and RNA transcription. Furthermore, the folate‐mediated one‐carbon metabolism pathway is induced to further support nucleotide biosynthesis. This pathway is found in both the cytosolic and mitochondrial compartments, and it has been established that key reactions take place in the mitochondria in order to produce the one‐carbon units which are necessary for the synthesis of nitrogenous bases [68]. Methylenetetrahydrofolate dehydrogenase 2 (MTHFD2) has been identified as a key player in this process, with recent studies indicating a correlation between MTHFD2 and the regulation of T‐cell fate and function [69]. Mitochondria are also the primary source of reactive oxygen species (ROS). During oxidative phosphorylation, electrons are transferred along the electron transport chain, generating a proton gradient that drives ATP synthesis. A small fraction of electrons can prematurely leak, resulting in ROS production [74]. Mitochondrial ROS exert both beneficial and detrimental effects on T‐cell biology [75, 76]. At physiological levels, ROS function as signalling molecules that enhance T‐cell receptor (TCR) activation and downstream signalling pathways, including nuclear factor kappa B (NF‐κB), nuclear factor of activated T cells (NFAT), and activator protein 1 (AP‐1), which are required for T‐cell activation, proliferation, and cytokine production [77, 78]. ROS also influence lineage commitment among CD4^+^ T‐cell subsets [75, 76]. Conversely, excessive ROS generation can promote T‐cell apoptosis, a process that may be relevant in specific pathological contexts [79]. In conditions of chronic inflammation or cancer, sustained ROS overproduction induces oxidative damage to lipids, proteins, and DNA, impairing T‐cell differentiation and function and ultimately compromising effective immune responses [80]. It is evident that this state contributes to the development of T‐cell exhaustion (Tex), a phenomenon associated with mitochondrial dysfunction, altered mitochondrial dynamics, and ROS accumulation. The phenomenon of T‐cell exhaustion has been observed in pathological states marked by persistent antigen stimulation, including chronic viral infections and neoplastic diseases [76, 77]. T cells, specifically CD8^+^ T cells, have been shown to demonstrate decreased effector function, diminished proliferative capacity, and augmented expression of inhibitory receptors such as programmed cell death protein 1 (PD‐1), lymphocyte activation gene 3 (LAG‐3), and T‐cell immunoglobulin and mucin‐domain containing‐3 (TIM‐3) [76]. This decline in function is characterised by the exhaustion of these vital cells, which can have significant consequences on overall health and immune response. The exhausted state of T cells is governed by distinct transcriptional and epigenetic programs. The regulatory function of these programmes is orchestrated by a specific set of transcription factors, comprising T‐bet (TBX21), B lymphocyte‐induced maturation protein 1 (BLIMP1), forkhead box O1 (FOXO1), thymocyte selection‐associated high mobility group box protein (TOX), NFAT, nuclear receptor subfamily 4 group A (NR4A), and eomesodermin (EOMES). Collectively, these factors contribute to the reshaping of chromatin accessibility and cellular metabolism [76, 77, 78]. Mitochondrial dynamics are profoundly altered during exhaustion; in particular, reduced dynamin‐related protein 1 (DRP1) activity has been observed in tumour‐infiltrating CD8^+^ T cells due to impaired phosphorylation at Ser616. This defect blocks mitochondrial fission, leading to the accumulation of hyperfused but dysfunctional mitochondria. DRP1 activity is further suppressed by PD‐1‐mediated signalling [81]. Through BLIMP1‐dependent mechanisms, PD‐1 signalling also inhibits peroxisome proliferator‐activated receptor gamma coactivator 1‐alpha (PGC‐1α), a master regulator of mitochondrial biogenesis [3]. As a consequence, damaged mitochondria accumulate and generate excessive ROS, further promoting oxidative stress and impairing TCR signalling. This redox imbalance favours pathological NFAT activation and induces exhaustion‐associated genes such as TOX and BLIMP1 [3, 12]. Notably, restoration of PGC‐1α expression has been shown to reverse PD‐1–mediated metabolic dysfunction and partially reinvigorate exhausted T cells [82].

Taken together, these observations support a model in which mitochondrial dysfunction participates directly in the establishment and maintenance of T‐cell exhaustion. The hypothesis suggests that persistent antigenic stimulation can initiate metabolic pressure. However, it is posited that defects in mitochondrial dynamics, biogenesis, redox control, and metabolite availability can subsequently reinforce exhaustion by reshaping transcription‐factor activity, chromatin accessibility, and lineage stability. Consequently, it is not merely a passive reporting mechanism for the energetic state of chronically stimulated T cells. Rather, mitochondria have the capacity to induce a state of dysfunction in these cells, and may also influence the reversal of such states through the implementation of therapeutic interventions.

In addition, T cells that have become exhausted continue to be replenished by a reserve of precursor or progenitor exhausted T cells. Accordingly, these progenitor or precursor exhausted T cells are, by definition, designated as “Tpre cells.” These cells can be identified through the expression of memory‐associated transcription factors, such as TCF‐1, and surface markers, including Ly108 and CD62L [81, 82, 83, 84]. Nevertheless, the mechanisms governing their maintenance and attrition remain incompletely understood. The utilisation of murine models in research endeavours has led to the identification of mitochondrial dysfunction as a pivotal regulator of this process. Mitochondrial alterations promote oxidative stress, which interferes with the degradation of HIF‐1α. Impaired HIF‐1α turnover subsequently drives transcriptional and metabolic reprogramming that favours the differentiation of Tpex cells into terminally exhausted T cells (Table 1). This pathway may be therapeutically exploitable in selected pathological settings to preserve or enhance Tpex cell function, for instance in the tumour microenvironment. This is particularly relevant because the balance between Tpex maintenance and terminal exhaustion may represent one of the most clinically meaningful readouts of mitochondrial fitness: when mitochondrial stress promotes HIF‐1α‐dependent glycolytic reprogramming, it may actively accelerate the loss of progenitor‐like potential rather than merely accompany terminal differentiation [10]. Although T‐cell exhaustion has been most extensively characterised in CD8^+^ T cells, recent evidence indicates that similar exhaustion‐like phenotypes can also occur in CD4^+^ T cells under pathological conditions [47, 83]. Indeed, CD4^+^ T‐cell dysfunction has been observed in chronic viral infections [84, 85] and cancer [86]. In chronic viral infection models, exhausted CD4^+^ T cells exhibit transcriptional features resembling those of exhausted CD8^+^ T cells, including the expression of BATF, TBX21, and PRDM1 [87].

A subsequent study concentrating on Th17 cells evidenced that, during differentiation, these cells underwent metabolic reprogramming, transitioning from an inaugural reliance on glycolysis to a subsequent stage distinguished by heightened reliance on mitochondrial oxidative phosphorylation (OXPHOS). It is noteworthy that Th17 cells displaying heightened mitochondrial activity, as evidenced by a hyperpolarised mitochondrial membrane potential (ΔΨ), exhibit diminished cytokine production. This observation suggests that accelerated early activation may render these cells vulnerable to functional exhaustion. This state is accompanied by the expression of exhaustion‐associated markers, including PD‐1 and TIGIT. As in other T‐cell subsets, these findings highlight a link between mitochondrial metabolism and functional fate and suggest potential opportunities for therapeutic intervention in pathological settings such as autoimmune diseases and cancer, in which Th17 cell activity contributes significantly to disease progression [88].

## 4 Pathological Contexts of T‐Cell Exhaustion and Mitochondrial Dysfunction

Mitochondrial dysfunction in T cells is considered by many to be a central mechanism underlying impaired immune responses, and it is widely accepted that it contributes to the development of a wide variety of pathological conditions (Table 1). Numerous studies have shown that defects in energy production, disturbances in redox homeostasis, and excessive reactive oxygen species (ROS) generation reshape T‐cell activation and metabolic programs in a range of disease settings, including cancer, ageing, and viral infections [28, 89]. A unifying feature of these conditions is that mitochondrial injury can act as a feed‐forward driver of immune dysfunction: once established, it may stabilise exhaustion‐ or senescence‐associated phenotypes and reduce the capacity of T cells to recover effective effector or memory responses. The exhaustion program described above provides the conceptual link between the cancer and chronic‐infection sections. In both settings, persistent antigen recognition, sustained inhibitory‐receptor signalling, inflammatory cues, and restricted access to oxygen or nutrients drive related trajectories from Tpex cells toward terminally exhausted states. The relative contribution of hypoxia, metabolite competition, viral products, and tissue‐specific inflammation differs between diseases, but mitochondrial depolarisation, defective biogenesis, altered fission–fusion balance, and excess reactive oxygen species are recurrent mechanisms that reinforce the shared transcriptional and epigenetic architecture of exhaustion [3, 10, 11, 12, 13, 47]. Accordingly, the following cancer and viral‐disease sections should be read as context‐specific manifestations of a common mitochondria–exhaustion axis, whereas ageing additionally involves senescence and inflammageing.

### 4.1 Mitochondrial Dysfunction and T‐Cell Exhaustion in Cancer

In tumours, this shared exhaustion pathway is intensified by microenvironmental hypoxia, nutrient competition, suppressive metabolites, and checkpoint signalling, which collectively limit mitochondrial renewal and accelerate progression from progenitor to terminally exhausted states [10, 11, 90]. Mitochondrial dysfunction has been shown to have a detrimental effect on the functioning of T cells in the tumour microenvironment (TME). Numerous studies, with a particular focus on CD8^+^ T lymphocytes, have identified the molecular and metabolic characteristics that underpin their defective responses to tumours in the presence of mitochondrial dysfunction [11]. It has been demonstrated that prolonged exposure of CD8^+^ T‐cells to the TME is associated with a reduction in PGC‐1α expression. This is a key regulator of mitochondrial biogenesis. PGC‐1α enables CD8^+^ T cells to adapt their mitochondrial network to the TME by supporting differentiation, metabolic fitness, organellar structure, and calcium transport [91]. In vitro studies under hypoxic conditions have shown that inhibition of PGC‐1α activity leads to CD8^+^ T‐cell depletion through increased ROS production. ROS accumulation inhibits phosphatases and activates the transcription factor NFAT, which in turn induces gene expression programs associated with T ‐cell exhaustion [90, 92]. Within the TME, activated CD8^+^ T cells partially adapt their metabolism by shifting towards glycolysis and glutaminolysis to sustain antitumour activity [11]. However, they must compete with tumour cells for essential nutrients such as glucose and oxygen, further compromising their metabolic capacity [90, 92, 93, 94]. Pyruvate generated by glycolysis normally fuels the tricarboxylic acid (TCA) cycle to support energy production, yet tumour cells restrict pyruvate availability, thereby limiting the energetic efficiency of CD8^+^ T cells [95]. In addition, loss of specific mitochondrial proteins, including PTPMT1, impairs pyruvate oxidation, reduces metabolic plasticity, and accelerates T ‐cell exhaustion in the TME [96]. The accumulation of lactate together with reduced availability of glucose and fatty acids further constrains CD8^+^ T‐cell effector function and promotes the establishment of an exhausted phenotype [90]. Consistent with these observations, T cells within the TME are frequently found in a state of functional exhaustion, which contributes to immune escape by tumours. Accordingly, increasing efforts are directed towards targeting mitochondrial metabolism in exhausted T cells to restore mitochondrial dynamics and partially recover antitumour immunity [97].

In parallel, immunosuppressive cells within the TME, including regulatory T cells (Tregs), tumour‐associated macrophages (TAMs), myeloid‐derived suppressor cells (MDSCs), and cancer‐associated fibroblasts (CAFs), further exacerbate T‐cell dysfunction by secreting inhibitory cytokines and chemokines and inducing the expression of immune checkpoint receptors such as PD‐1, CTLA‐4, LAG‐3, and TIM‐3 [90]. These findings provided the rationale for the development and clinical application of immune checkpoint inhibitors, including anti–PD‐1 antibodies, in the treatment of multiple cancer types [98].

Mitochondria also participate in a process known as mitochondrial transfer, whereby intact mitochondria or mitochondrial components are exchanged between cells. Typically, this mechanism enables donor cells to supply functional mitochondria to recipient cells with damaged organelles, thereby restoring metabolic competence and supporting cell survival. Mitochondrial transfer contributes to tissue repair and immune regulation but is also implicated in cancer and neurodegenerative diseases. It occurs through specialised structures such as tunnelling nanotubes (TNTs), extracellular vesicles, or cell–cell interactions including endocytosis and reverse phagocytosis [99]. Within the TME, however, this process can be co‐opted by tumour cells as a strategy to evade immune control [100]. Oxidative stress plays a central role in regulating mitochondrial transfer, as ROS generated during mitochondrial respiration influence the trafficking of intracellular organelles [101]. Preliminary clinical findings suggest that mitochondrial transfer from tumour cells to tumour‐infiltrating lymphocytes (TILs) can modulate their biological state, influencing their metabolic profile and potentially inducing cellular senescence [102]. Moreover, computational modelling employing the MERCI framework posits the possibility that this exchange may be unidirectional, manifesting predominantly from immune cells to tumour cells. This phenomenon has the potential to exacerbate tumour metabolism, which may in turn impair T‐cell functionality [103]. This mechanism strongly shapes antitumour immunity but may also represent a therapeutic target, as modulating or reversing mitochondrial transfer between tumour cells and T lymphocytes could help restore immune competence within the TME [100].

### 4.2 Mitochondrial Dysfunction During the Ageing Process

During physiological ageing, multiple biological processes undergo progressive alterations in cellular function, leading to a decline in immune competence and reduced protection against infections and cancer. This immune impairment reflects both intrinsic defects in immune effector cells and the presence of a chronic low‐grade inflammatory state, commonly referred to as “inflammageing” [104]. Mitochondrial dysfunction is a hallmark of the ageing process. This condition is characterised by the presence of mutations in mitochondrial DNA (mtDNA), a decline in respiratory capacity, a decrease in membrane potential, and an increase in the production of reactive oxygen species (ROS). The aforementioned changes compromise mitochondria‐associated processes, including but not limited to cellular metabolism, mitochondrial dynamics and biogenesis, mitophagy, calcium homeostasis, and apoptotic signalling [105]. T lymphocytes, which play a pivotal role in the formation of immunological memory, demonstrate a reduced ability to discern and react to antigens. This decline is attributable to alterations in metabolic pathways and mitochondrial dysfunction, resulting in elevated oxidative stress and subsequent deterioration of T‐cell functionality. In individuals with age‐associated diseases, increased mtDNA mutation rates are frequently observed. In particular, reduced expression of MRE11A, a nuclease involved in DNA repair, in CD4^+^ T cells results in impaired mtDNA stability, heightened oxidative stress, and altered oxidative phosphorylation [106]. mtDNA instability promotes the release of mitochondrial DNA into the cytosol, activating innate inflammatory pathways such as cGAS–STING, which induces type I interferon and pro‐inflammatory cytokine production, thereby amplifying ROS levels [102], as well as the NLRP3 inflammasome, which triggers pyroptosis through IL‐1β and IL‐18 [103,105]. Activation of these pathways exacerbates mitochondrial dysfunction and promotes acute inflammatory responses associated with declining T‐cell function in ageing, as well as in pathological conditions such as HIV infection and rheumatoid arthritis [107].

Additional mitochondrial‐derived damage‐associated molecular patterns (mtDAMPs) further contribute to inflammageing. For example, cardiolipin, which is required for cristae curvature, facilitates inflammasome assembly [105], while SMAC, a regulator of apoptotic signalling, modulates NF‐κB activity and can promote the reprogramming of TH17 cells toward a TH2 phenotype [108, 109]. Other metabolic factors also participate in age‐related T‐cell decline, including imbalances in the NAD^+^/NADH ratio and NAD^+^ depletion driven by CD38 and PARP1 activity, both of which impair mitochondrial function [110, 111]. Moreover, a pro‐inflammatory milieu sustained by ROS, DAMPs, and cytokines such as IL‐6 and IFN‐γ released by senescent cells as part of the senescence‐associated secretory phenotype (SASP) further disrupts mitochondrial dynamics and function, establishing a feed‐forward loop that accelerates T ‐cell dysfunction during ageing [112, 113, 114]. It has been demonstrated that, with the passage of time, the accumulation of dysfunctional mitochondria promotes the emergence of T cells associated with ageing (T cells, or TAA, for short). TAAs are known to contribute to several serious conditions, including chronic inflammation, autoimmunity, susceptibility to infections, and cancer progression. These findings provide a rationale for the development of therapeutic strategies aimed at preserving mitochondrial integrity and T‐cell function in older individuals with the objective of mitigating immune decline associated with ageing [115].

### 4.3 Mitochondrial Dysfunction and T‐Cell Exhaustion in Chronic Viral Diseases

Chronic viral infection engages a closely related differentiation hierarchy: persistent antigen sustains Tpex cells but progressively favours terminal exhaustion when mitochondrial damage, oxidative stress, and bioenergetic insufficiency accumulate. Unlike tumours, viral diseases also introduce pathogen‐specific effects and treatment‐related influences, yet the core coupling between mitochondrial failure and exhaustion remains conserved [12, 13, 47].

Over recent years, multiple studies have documented the presence of mitochondrial dysfunction in T cells from patients with viral infections. In the context of HIV infection, significant mitochondrial alterations have been documented, impacting immune cell metabolism, particularly in CD4^+^ and CD8^+^ T cells and macrophages. These alterations contribute to immune impairment and chronic inflammation [116]. As has been noted, the mitochondrial function of CD4^+^ lymphocytes is particularly compromised in individuals receiving effective antiretroviral therapy (ART) who fail to achieve complete immune reconstitution, categorised as immune non‐responders (INRs) [117]. In the patients examined, CD4^+^ T cells exhibited persistent activation and exhaustion, along with features of cellular senescence. Concurrently, mitochondrial aberrations were observed, including reduced respiratory capacity, altered oxidative phosphorylation (OXPHOS), and accumulation of mtDNA damage [118]. It has been demonstrated that the expression of mitochondrial transcription factor A (mtTFA), an essential component in the maintenance and replication of mitochondrial deoxyribonucleic acid (mtDNA), exhibits a significant decrease in individuals infected with HIV in comparison with healthy control subjects. Restoration of mtTFA expression in CD4^+^ T cells from HIV‐infected patients improves mitochondrial function, indicating that targeting the mtTFA‐dependent pathway may represent a therapeutic strategy to enhance immune recovery, particularly in INRs [119].

In HIV‐infected cells, increased ATP production has also been reported. Mitochondrial dysfunction, associated with enhanced OXPHOS activity during latent infection, promotes upregulation of the pannexin‐1 channel and the consequent release of ATP into the extracellular space [115, 120]. Extracellular ATP activates purinergic P21 and P2x7 receptors expressed by immune cells [63], especially T lymphocytes, thereby reinforcing the pro‐inflammatory environment characteristic of HIV infection [115, 120, 121]. As observed during ageing, mitochondrial dysfunction also promotes ROS generation and the release of oxidised mtDNA, leading to activation of the NLRP3 inflammasome. This pathway contributes to CD4^+^ T‐cell loss, neuroinflammation, and HIV‐associated neurocognitive disorders [120, 122]. Furthermore, the presence of NLRP3‐driven inflammation has been associated with an augmented risk of cardiovascular disease among individuals living with HIV who are undergoing antiretroviral therapy (ART) and manifest elevated levels of IL‐6, high‐sensitivity C‐reactive protein (hsCRP), and D‐dimer [123].

In recent studies, mitochondrial dysfunction has been associated with the lymphocytopenia observed in patients with severe SARS‐CoV‐2 infection. Lymphocytes from these individuals demonstrate significant mitochondrial alterations, including impaired oxidative phosphorylation and elevated oxidative stress. This results in defective T‐cell activation, proliferation, and antiviral responses [118]. Consistent with observations in cancer, viral infections are also associated with CD8^+^ T‐cell exhaustion. During chronic hepatitis B virus (HBV) infection, for example, sustained viral persistence drives progressive CD8^+^ T‐cell dysfunction and loss of effector capacity, a process that is closely linked to deterioration of mitochondrial function [13].

## 5 Therapeutic Strategies for Restoring T Cell Function in Pathological Contexts

As discussed above, mitochondrial dysfunction appears to be a key contributor to impaired T‐cell function in several pathological contexts and may therefore represent a potential target for therapeutic intervention aimed at restoring immune competence (Table 1). Within the tumour microenvironment, characterised by nutrient deprivation and hypoxia, and in chronic viral infections associated with sustained immunosuppressive signalling, T cells progressively lose mitochondrial fitness, which is associated with the development of functional exhaustion [124, 125]. This condition is commonly accompanied by reduced oxidative phosphorylation, increased reactive oxygen species (ROS) production, and upregulation of inhibitory receptors such as PD‐1 and TIM‐3 [97]. Accordingly, a number of therapeutic strategies have been proposed to modulate mitochondrial metabolism in exhausted T cells and improve their functional capacity [3]. Enhancement of mitochondrial biogenesis has been suggested as a possible approach to limit T‐cell exhaustion and support T‐cell function. In particular, activation of the transcriptional coactivator PGC‐1α has been shown to promote mitochondrial biogenesis and respiratory capacity, favour memory T‐cell formation, and improve the function of exhausted CD8^+^ T cells [13, 126]. Pharmacological or molecular agents that stimulate PGC‐1α activity, including complement‐binding protein (C1QBP), metformin, bezafibrate, and IL‐15, especially when combined with immune checkpoint blockade (e.g., anti–PD–1 antibodies), have been reported to improve mitochondrial metabolism and may enhance the efficacy of immunotherapy [123, 127, 128, 129]. In addition, co‐stimulation through 4‐1BB and supplementation with coenzyme Q10 (CoQ10) have been associated with improved mitochondrial performance by supporting biogenesis and fusion, potentially counteracting features of T‐cell exhaustion [130, 131]. Excessive ROS production is also thought to contribute to T‐cell dysfunction. Several studies suggest that reducing oxidative stress can partially restore T ‐cell activity [3]. Antioxidants such as N‐acetylcysteine (NAC) and mitochondria‐targeted compounds including MitoQ, MitoTempo, and Trolox have been shown to improve mitochondrial capacity and attenuate exhaustion‐associated phenotypes [13, 132, 133]. Restoration of intracellular NAD^+^ levels through nicotinamide riboside (NR) or nicotinamide (NAM) has likewise been reported to reduce oxidative stress and support mitochondrial function [3, 134, 135]. During chronic infection, ATP depletion may further compromise T‐cell responses by impairing mitochondrial metabolism. In this setting, inhibition of the CD39/CD73 axis, which converts extracellular ATP into adenosine, has been proposed as a strategy to enhance immune activity and limit T‐cell exhaustion [136, 137, 138]. Moreover, loss of key regulators of ATP production and mitochondrial dynamics, such as USP25 or ATP5IF1, has been implicated in T‐cell dysfunction [139, 140], whereas their restoration has been associated with improved antitumour immunity [141]. Furthermore, the notion of mitochondrial transfer has emerged as a promising avenue for the restoration of mitochondrial fitness in exhausted T cells [142]. The process under consideration may enable delivery of functional mitochondria to a cohort of T cells that are metabolically compromised. The result of this process may be the metabolic reprogramming and functional recovery of the T cells. Recent studies suggest that tunnelling nanotubes (TNTs) can facilitate unidirectional and bidirectional mitochondrial transfer between stromal cells and T lymphocytes, thereby enhancing mitochondrial capacity and potentially improving effector function [3, 143]. The therapeutic implication of this finding is considerable; if mitochondrial defects are indeed the underlying cause of exhaustion, then interventions aimed at restoring mitochondrial function could lead not only to improved bioenergetics but also to the reopening of otherwise constrained T‐cell differentiation pathways and functional programs. Consequently, strategies aimed at enhancing mitochondrial integrity by promoting mitochondrial biogenesis, constraining oxidative stress, or facilitating mitochondrial transfer have the potential to establish novel therapeutic methodologies designed to reinvigorate dysfunctional T cells. Such interventions could contribute to reducing immune deficits and improving both antitumour and antiviral immune responses. Thus, mitochondrial rescue should be viewed not simply as metabolic support, but as a strategy to alter the stability and reversibility of dysfunctional T‐cell states.

## 6 Conclusion

In conclusion, the studies discussed throughout this review support the view that mitochondria are not merely bioenergetic “support units” in T cells, but rather dynamic hubs that integrate metabolism with signalling, differentiation, and long‐term immune fitness [70]. Across cancer, ageing, and chronic viral infections, a recurring theme is that loss of mitochondrial integrity, whether through disrupted biogenesis, altered dynamics, redox imbalance, or mtDNA instability, tends to coincide with reduced functional plasticity and the gradual emergence of exhaustion‐ or senescence‐like states [13, 112, 117, 144, 145]. At the same time, the field is beginning to converge on the idea that mitochondrial defects are not simply bystanders of chronic inflammation or persistent antigen exposure but may actively shape the transcriptional and epigenetic programs that lock T cells into dysfunctional trajectories [47]. This raises an important, and still open, question: to what extent mitochondrial “recovery” can meaningfully reset T‐cell fate once exhaustion is established, and which mitochondrial nodes are most amenable to intervention without compromising physiological immune activation [3, 13]. Current evidence indicates that strategies aimed at enhancing mitochondrial fitness through approaches such as promoting PGC‐1α‐driven biogenesis, mitigating oxidative stress, restoring NAD^+^ levels, modulating purinergic signalling, or facilitating mitochondrial transfer can promote more favourable metabolic and functional states, particularly in conjunction with established immunotherapies like checkpoint blockade [3]. In future studies, it will be crucial to establish the context‐dependency of these strategies, identify robust biomarkers of mitochondrial competence in T‐cell subsets (including Tpex populations), and elucidate the causal relationships between mitochondrial remodelling and immune outcomes. A more mechanistic understanding of how mitochondrial structure, metabolism, and dynamics are coordinated with immune signalling should ultimately help translate these concepts into targeted interventions that preserve or reinvigorate T‐cell function in disease.

Overall, the central message emerging from this review is that mitochondrial dysfunction should be interpreted as an active determinant of T‐cell fate rather than as a secondary metabolic consequence of chronic immune stimulation. By influencing redox signalling, metabolite‐dependent epigenetic regulation, organelle dynamics, and biogenesis, mitochondria can contribute to the stabilisation of exhausted or senescent phenotypes. Conversely, the same mitochondrial nodes may define therapeutic reversibility, identifying mitochondrial fitness as both a biomarker of T‐cell competence and a targetable axis for restoring immune function.

## Author Contributions

**Rosario Amato**: designed the study. **Carolina Brescia and Rosario Amato**: drafted the manuscript. **Carolina Brescia, Giuseppe Viglietto and Rosario Amato**: reviewed the article. All authors read and approved the final version of the manuscript.

## Conflicts of Interest

The authors declare no conflicts of interest.

## Data Availability

Data sharing is not applicable to this article, as no datasets were generated or analysed during the current study.
